# Epigenetic insights of Olympic champions: nuclear and mitochondrial DNA methylation and regulators of aging

**DOI:** 10.1007/s11357-025-02092-9

**Published:** 2026-01-17

**Authors:** Timea Teglas, Ferenc Torma, Zoltan Bori, Dora Aczel, Gergely Babszky, Takuji Kawamura, Mitsuru Higuchi, Gu Yaodong, Muhammad Lee, Steve Horvath, Zsolt Radak

**Affiliations:** 1 Research Center for Molecular Exercise Science, Hungarian University of Sports Science, Alkotás U. 42-48, 1123 Budapest, Hungary; 2 Faculty of Sport Sciences, Waseda University, Tokorozawa, Japan; 3 Faculty of Sport Science, Ningbo University, Ningbo, China; 4 Asian College of Sport Medicine, Petaling Jaya, Malaysia; 5 Department of Biostatistics, Fielding School of Public Health, University of California, Los Angeles, Los Angeles, CA USA; 6 Altos Labs, Cambridge, Cambridgeshire UK

**Keywords:** Aging, Olympic champions, Mitochondrial DNA, Methylation

## Abstract

The interaction between nuclear (nDNA) and mitochondrial DNA (mtDNA) methylation is not well known in the healthy population. The D-loop methylation level of the Olympic champions (*N* = 58) was significantly lower than that of non-champions (*N* = 32) (~ 36% unadjusted mean difference *p* = 0.016, sex and age adjusted *p* = 0.017). Interestingly, the robust linear analysis revealed that biological sex is a significant factor in mtDNA D-loop methylation (estimate = 1.521, *p* = 0.033). On the other hand, we cannot find relationships between the methylation levels of mtDNA and nuclear DNA, suggesting distinct regulation of the methylation/demethylation process of mtDNA and nuclear DNA. DNA methylation-based aging clocks showed a significant relationship with the levels of Klotho, irisin, and its receptor (irisin receptor integrin alpha-V), as well as with epigenetic regulators such as ten-eleven translocation enzyme 2, which were measured using enzyme-linked immunosorbent assay. Therefore, the data suggest a complex regulatory process of epigenetic aging and raise the possibility that D-loop methylation may have functional relevance in health, which remains to be explored.

**Graphical abstract:**

**Figure:**
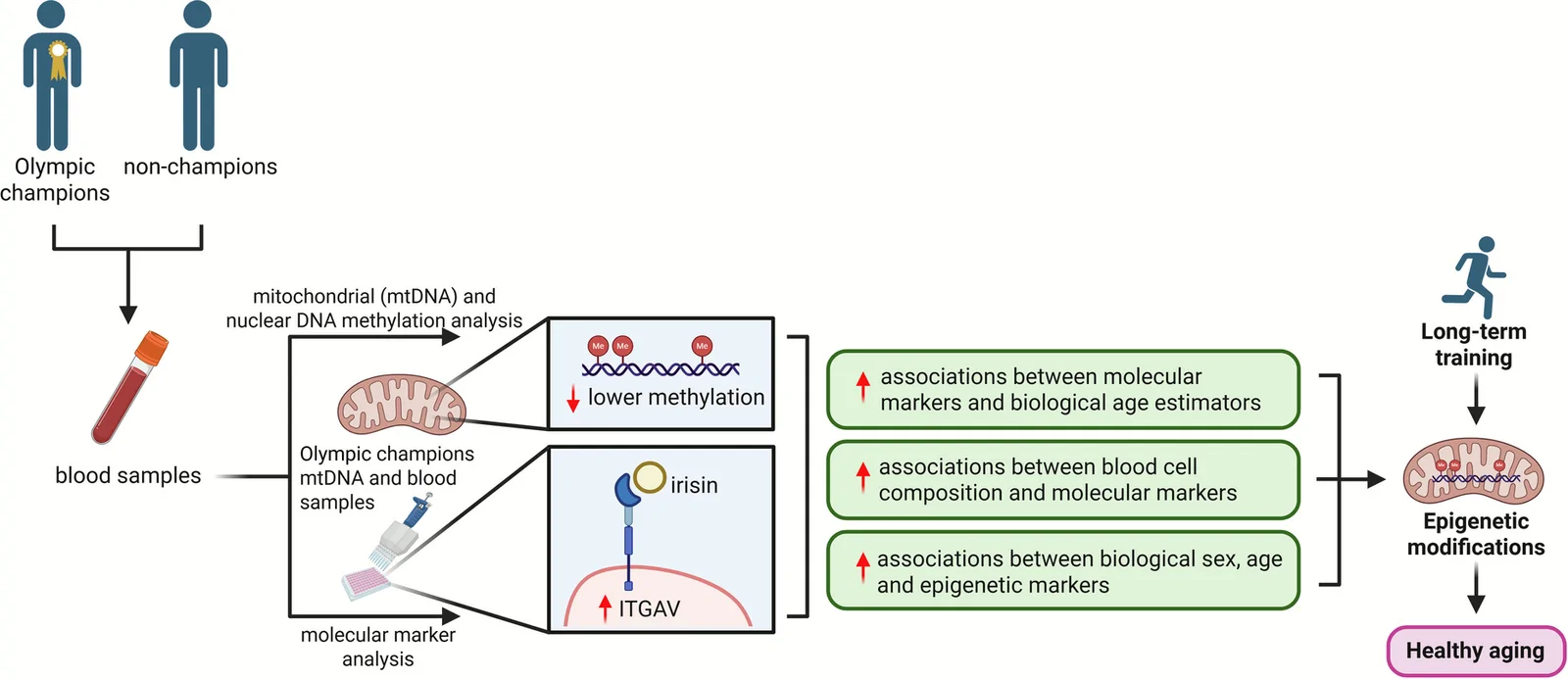

**Supplementary Information:**

The online version contains supplementary material available at https://doi.org/10.1007/s11357-025-02092-9.

## Introduction

DNA is regularly wrapped around histone proteins in the nucleus, shaping packaged units called nucleosomes. The methylation and demethylation of CpG islands tighten or relax the chromatin that influences gene expression [1]. The promoter region of genes is rich in CpG islands, and the family of DNA methyltransferases inserts methyl groups into these regions. The DNA demethylation mechanism is found to be mediated by the ten–eleven translocation (Tet) enzymes. The location and dynamics of DNA methylation can alter gene expression and are integral to epigenetic regulation.

The methylation of mtDNA occurs at a significantly lower degree than that of nuclear DNA. The D-loop region of the mtDNA is essential to controlling its replication and transcription, and the methylation of the D-loop is associated with diseases [2]. Indeed, it has been reported that methylation of mtDNA is associated with obesity and an obesity-related phenotype [3]. However, it has also been reported that resistance training alters mtDNA methylation in human skeletal muscle, which could be associated with enhanced transcription and rejuvenation [4]. Additionally, it is known that the methylation levels of the D-loop are associated with oxidative stress, particularly with the accumulation of 8-oxoG [5], which is linked to mitochondrial dysfunction. Indeed, mitochondrial dysfunction and DNA methylation are listed among the hallmarks of aging [6]. The methylation characteristics of nuclear DNA have been utilized to develop epigenetic aging clocks, which estimate aging progress and predict mortality and disease risk. However, the potential interaction between mtDNA and nuclear DNA methylation patterns is poorly understood, especially in healthy populations.

Regular exercise is often mentioned as a natural means to delay the progression of aging. Indeed, it is also known that a higher level of physical fitness is associated with a decreased aging process, as evaluated with DNA methylation-based aging clocks [7, 8]. A vast amount of epidemiological observation suggests that a higher level of physical fitness decreases the incidence of a wide range of diseases [9, 10].

Due to the systemic effects of exercise, several contributing mechanisms and pathways are linked to age deceleration or healthy aging. Deficiency of the Klotho gene and the encoded circulating hormonal proteins is associated with aging phenotypes, including loss of muscular performance and enhanced sarcopenia [11, 12]. Higher level of physical fitness is associated with higher levels of circulating Klotho proteins [13, 14].

There are conflicting results on the exercise-mediated effects of circulating irisin, a myokine readily induced by exercise via fibronectin type III domain containing 5 (FNDC5) [15–17]. Irisin is associated with increased circulating brain-derived neurotrophic factor (BDNF) levels [18] and better cognitive function [19]. Moreover, the result of the meta-analysis suggests that subjects with sarcopenia have a lower level of irisin than their healthy counterpart [20]. It is suggested that irisin uses the αV integrins as a receptor [21].

Our recent investigation demonstrated that Olympic champions (aged 25–102 years) exhibit slower DNA–methylation–based aging clocks compared with recreational athletes and sedentary individuals [22] suggesting that long-term engagement in regular high-intensity exercise may contribute to decelerated biological aging. Previous studies on elite athletes have yielded mixed results. For example, Spólnicka et al. (2018) reported accelerated methylation age in some elite athletes, based on a 5-CpG model [23]. In contrast, a recent and larger study on 59 Olympic champions demonstrated slower epigenetic aging compared to controls [22]. Moreover, regular physical fitness has been associated with a younger biological age when using more comprehensive epigenetic clocks such as DNAmFitAge [19]. Finally, investigations in professional soccer players showed that even acute bouts of strenuous activity could transiently affect epigenetic clock readings [24]. Although these findings highlight a clear association between athletic excellence and epigenetic age dynamics, the underlying biological processes driving this phenomenon remain to be elucidated. In particular, the potential relationships between mtDNA methylation and nuclear DNA methylation—as well as circulating Klotho and irisin levels—are still unknown and have not yet been systematically investigated. In the present study, we aim to investigate these relationships and provide deeper insight into their potential biological relevance.

## Materials and methods

### Study design and subjects

The study included 90 participants, divided into two groups: an Olympic champion group (*n* = 58) and an age-matched non-athlete group (*n *= 32). The participants in the group of Olympic champions had retired from professional sports. Subject characteristics and distributions are depicted in Supplementary Fig. 1. The chronological age of the Olympic champions and non-athletes did not differ significantly. Participants completed a questionnaire that included demographic information as well as questions regarding their health status, smoking habits, medication use, and the presence of any medical conditions. Supplementary Fig. 2 presents the distribution of responses, chronic disease status, medication use, and smoking habits of the participants. The distribution of sports and years since career termination among Olympic champions is depicted in Supplementary Fig. 3.

### Blood collection

After completing the questionnaire, venous blood was drawn into K3EDTA (#484511, Greiner Bio-One GmbH, Austria) and CAT serum separator tubes (#485502, Greiner Bio-One GmbH, Austria) from each study participant. Plasma samples in the K3EDTA tubes were centrifuged at 1600 × g for 15 min at 4 °C, and the supernatant was collected. For serum, the samples in tubes with serum clot activator were incubated for 30 min at room temperature, then centrifuged at 3000 rpm for 10 min, and the upper, separated part of the blood was collected. For irisin analysis, 50 µL aprotinin (#A6279, Sigma-Aldrich) was added to 450 µL plasma samples. All the samples were stored at − 80 °C until the ELISA tests were performed or analyzed for blood biochemistry.

### ELISA analysis

ELISA kits were used to measure α-Klotho (SAKL, #EH4278), irisin receptor integrin alpha-V (ITGAV, #EH0815), and a methylcytosine dioxygenase ten-eleven translocation enzyme 2 (TET2, #EH12901). The ELISA kits were purchased from FineTest Biotech Inc. (Boulder, CO, USA). To measure the exercise response protein, irisin ELISA kits were purchased from Phoenix Pharmaceuticals Inc. (#EK-067-29). The procedures followed the manufacturers’ instructions, and the findings were measured using a Thermo Labsystems Multiskan EX plate reader (Thermo Fisher Scientific).

### Extraction of genomic DNA and bisulfite modification

For genomic DNA extraction, the whole blood (K3EDTA tubes) was used from each sample. Genomic DNA was extracted using the PureLink Genomic DNA Mini Kit (ThermoFisher Scientific, USA, Catalog #K182002) according to the manufacturer’s protocol. The extracted DNA was quantified using a DS-11 FX spectrophotometer/fluorometer (DeNovix, USA). After quantification, 500 ng of DNA from each sample was treated with sodium bisulfate to convert all unmethylated cytosines into uracil. The EZ DNA Methylation-Gold Kit (Zymo Research, USA, #D5005) was used for this procedure with 500 ng input DNA extract, following the manufacturer’s instructions. The bisulfide-modified DNA was eluted in 100 μL elution buffer. The Human Methylated and Non-methylated DNA Set (Zymo Research, USA, #D5014) was used as controls to verify the efficiency of the bisulfite conversion, with an average conversion rate of greater than 99%.

### Methylation sensitive-high resolution melting (MS-HRM) analysis

In the methylation-sensitive high-resolution melting (MS-HRM) analysis, each kit was used to treat simultaneously the same number of Olympic champion and control samples to avoid potential batch effects. Furthermore, samples derived from different bisulfite treatments were analyzed independently on separate occasions to verify the inter-assay variability. Methylation of the mitochondrial DNA D-loop region was assessed using the MS-HRM protocol with a QuantStudio™ 3 Real-Time PCR system and HRM Software v3.2 (Applied Biosystems, USA). The methylation-independent primers (MIPs) were designed using the software MethPrimer and reaction conditions based on the publication by Stoccoro et al. [25]. The sequences of the primers were as follows: forward, 5′-GGAGTTTTTTATGTATTTGGTATTTT-3′, and reverse, 5′-ACAAACATTCAATTATTATTATTATATCCT-3′. The amplified D-loop amplicon, spanning 222 bp from 35 to 256 nt (GenBank accession, #J01415.2), included 10 CpG sites. The standard conditions for the MS-HRM PCR were: 10 µL Precision Melt Supermix (Bio-Rad, USA), 10 pmol of each primer, 2 µL of EvaGreen® dye 20 × in water (Biotium, USA), and 5 µL of bisulfide-modified DNA eluates (equivalent to 25 ng of original DNA) in a total volume of 20 µL. The PCR temperature profile was as follows: 95 °C for 12 min, 95 °C for 30 s, 56 °C for 45 s, 72 °C for 30 s (Repeated steps 2, 3, and 4 40 times), and 72 °C for 10 min. The HRM temperature profile was the following: 95 °C for 10 s, 50 °C for 1 min, 65 °C for 15 s, dissociation step 0.015 °C/s (to 95 °C) with continuous data collections and final step 60 °C for 15 s.

### Measurement and quality control of nuclear DNA cytosine methylation

DNA cytosine methylation was measured using the Illumina Infinium MethylationEPIC platform (Illumina Inc., San Diego, CA), as previously described [22]. Briefly, DNA was extracted from EDTA-containing whole blood samples and subsequently converted to bisulfite using the EZ-96 DNA Methylation MagPrep Kit (Zymo Research, Irvine, CA, USA) according to the manufacturer’s protocol. A total of 8 µL of bisulfite-treated DNA was hybridized onto the EPIC BeadChip, and the arrays were processed according to Illumina’s standard protocol. Data quality control was performed using the minfi, Meffil, and ewastools packages in the R 4.4.1 software environment. Background correction was applied using the normal-exponential out-of-band (“noob”) normalization method [26]. Low-quality and unstable probes were filtered out based on a detection *p*-value threshold of greater than 0.01, resulting in a final dataset of 729,870 probes for subsequent analysis.

### Epigenetic biomarkers of ageing and blood cell composition estimation

DNA methylation-based biological age estimators were calculated using the DNA Methylation Age Calculator developed by the Clock Foundation Team (https://dnamage.clockfoundation.org/clock). The estimators included DNAmFitAge [27], and the robust versions of PhenoAge [28], GrimAge [29, 30], Horvath’s pan-tissue clock [31], Hannum blood-based clock [32], and Horvath’s SkinBlood clock [33]. MethylCIPHER R package is utilized to implement robust epigenetic clocks. Robust clocks are modified versions of the original clocks that incorporate additional statistical methods to enhance reliability and mitigate potential biases resulting from technical and biological variations. These principal component–based clocks [34] provide more robust longitudinal reliability. In addition to epigenetic clocks, blood cell composition was estimated from the DNA methylation data and used as a potential confounding variable by applying the Houseman method [35] and the EpiDish R package [36, 37].

### Statistical analysis

Statistical analyses were conducted using R version 4.4.1, with the ppcor and robustbase packages employed. Because many variables exhibited non-normal distributions, Spearman’s correlation was used for association analyses and ELISA results. In addition, log(e)-transformation was applied to stabilize the variance. For covariate-adjusted models, robust estimation methods were employed, using MM-type estimators for linear regression models [38, 39]. Epigenome-wide association studies (EWAS) were conducted by using the cpgassoc R package [40]. For the EWAS, we used ln-transformed circulating ITGAV levels and mtDNA D-loop methylation (measured MS-HRM) as the phenotypic outcome variables, while the predictors were nuclear DNA methylation levels measured with the Illumina EPIC array. Mixed-effects models were applied to test associations between each CpG site and the two outcomes, with chronological age, biological sex, and estimated blood cell composition included as fixed effects. Chip ID was incorporated as a random effect to account for array-specific technical variation. Multiple testing correction was addressed by applying a significance threshold of *p* < 9 × 10^−^⁸, as recommended by Mansell et al. [41].

## Results

### DNA methylation in Olympic champions and non-athlete individuals

**Fig. 1:**
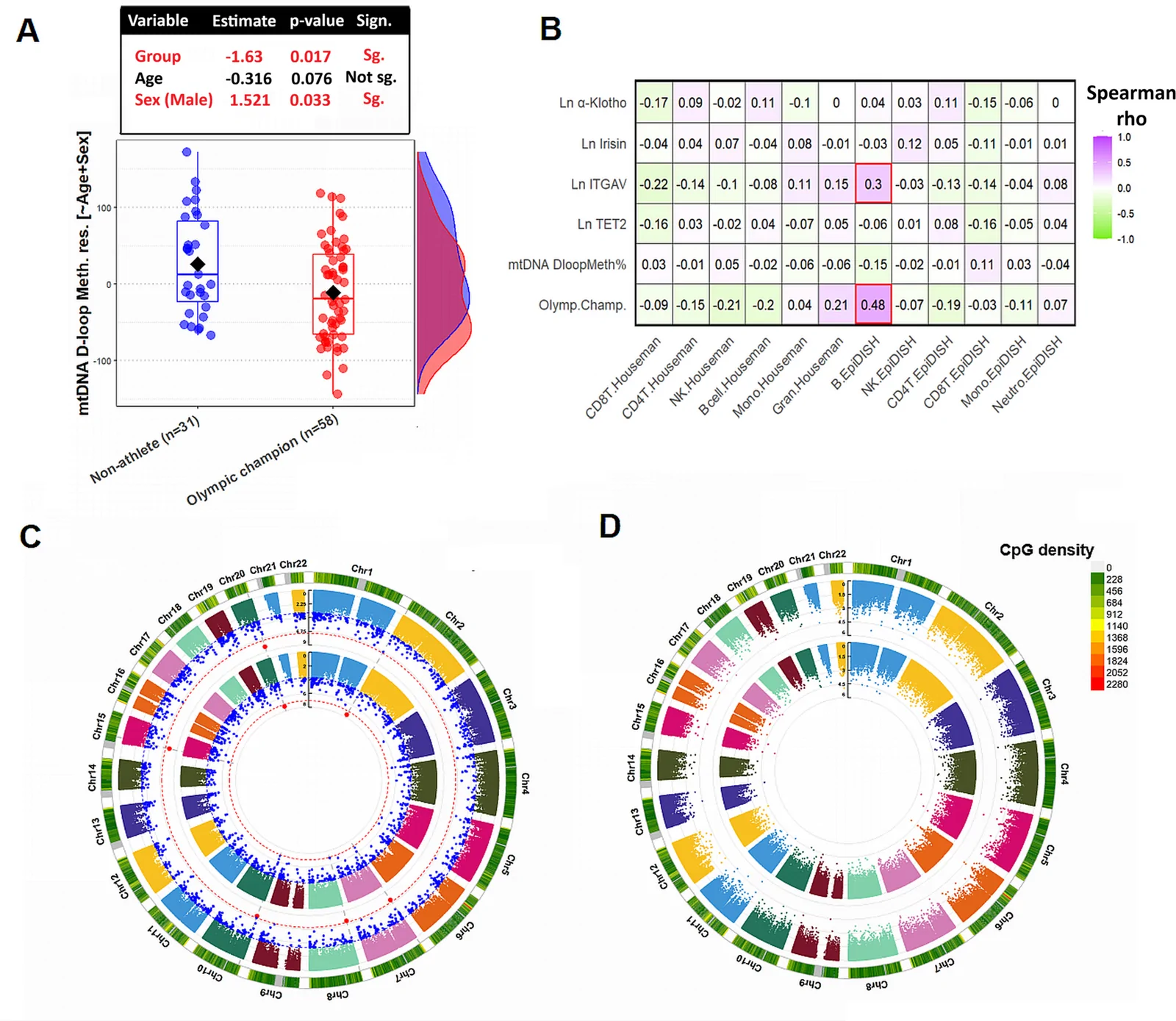
**A** mtDNA D-loop methylation group differences after adjustment for age and biological sex using robust linear models. **B** Correlation of estimated blood cell components with protein- and methylation-based markers. **C** EWAS results for ln-transformed ITGAV and **D** mtDNA D-loop methylation (measured by MS-HRM). **C** and **D** Associations between individual CpG sites and the respective outcome using circular Manhattan plots, where each point represents a single CpG site. The outer circle shows unadjusted associations, and the inner circle shows associations adjusted for age, sex, and blood cell composition. Points above the blue dashed line indicate FDR-significant associations, while points above the red dashed line indicate associations reaching the genome-wide significance threshold (*p* < 9 × 10⁻⁸). Sg.=significant effect

The D-loop methylation level of the Olympic champions was significantly lower (unadjusted ~36% mean difference, *p* = 0.016, sex and age adjusted *p* = 0.017) than non-champions (Fig. 1A). Interestingly, the robust linear analysis revealed that biological sex is a significant factor in mtDNA D-loop methylation (estimate = 1.521, *p* = 0.033).

On the other hand, we cannot find relationships between the D-loop overall methylation levels of mtDNA and nuclear genomic DNA (Fig. 1D), suggesting distinct regulation of the methylation/demethylation process of mtDNA and nuclear DNA. According to our analyses, neither mitochondrial DNA D-loop methylation (Fig. 1D) nor TET2 levels showed (data not shown) a significant association with individual nuclear cytosine methylation beta values in our sample.

In contrast, ITGAV levels, after technical adjustment, showed five significant associations following correction for multiple comparison errors. These CpG sites include cg22577284, cg02575956, cg14347424, cg00196715, and cg13649332, corresponding to the APCDD1L-AS1 (gene body), AQR (gene body), PRKAR1B (TSS1500 region), SFMBT2 (gene body), and DLGAP2 (gene body) genes (Fig. 1C outer circle). After cell type correction, only the probes cg21465236 (MYT1L) and cg22577284 (APCDD1L-AS1) remained significantly associated with ln ITGAV levels (Fig. 1C, inner circle).

To gain insight into biological relevance, we tested whether any Gene Ontology terms were significantly enriched among the CpG probes associated with ln-transformed ITGAV at a false discovery rate (FDR) of less than 0.1. In our sample, no significant enrichment was detected using the gometh function of the missMethyl R package [42], which accounts for the differing number of probes per gene present on the beadchip array (Supplementary Fig. 4).

### Differences in molecular markers in Olympic champions

**Fig. 2:**
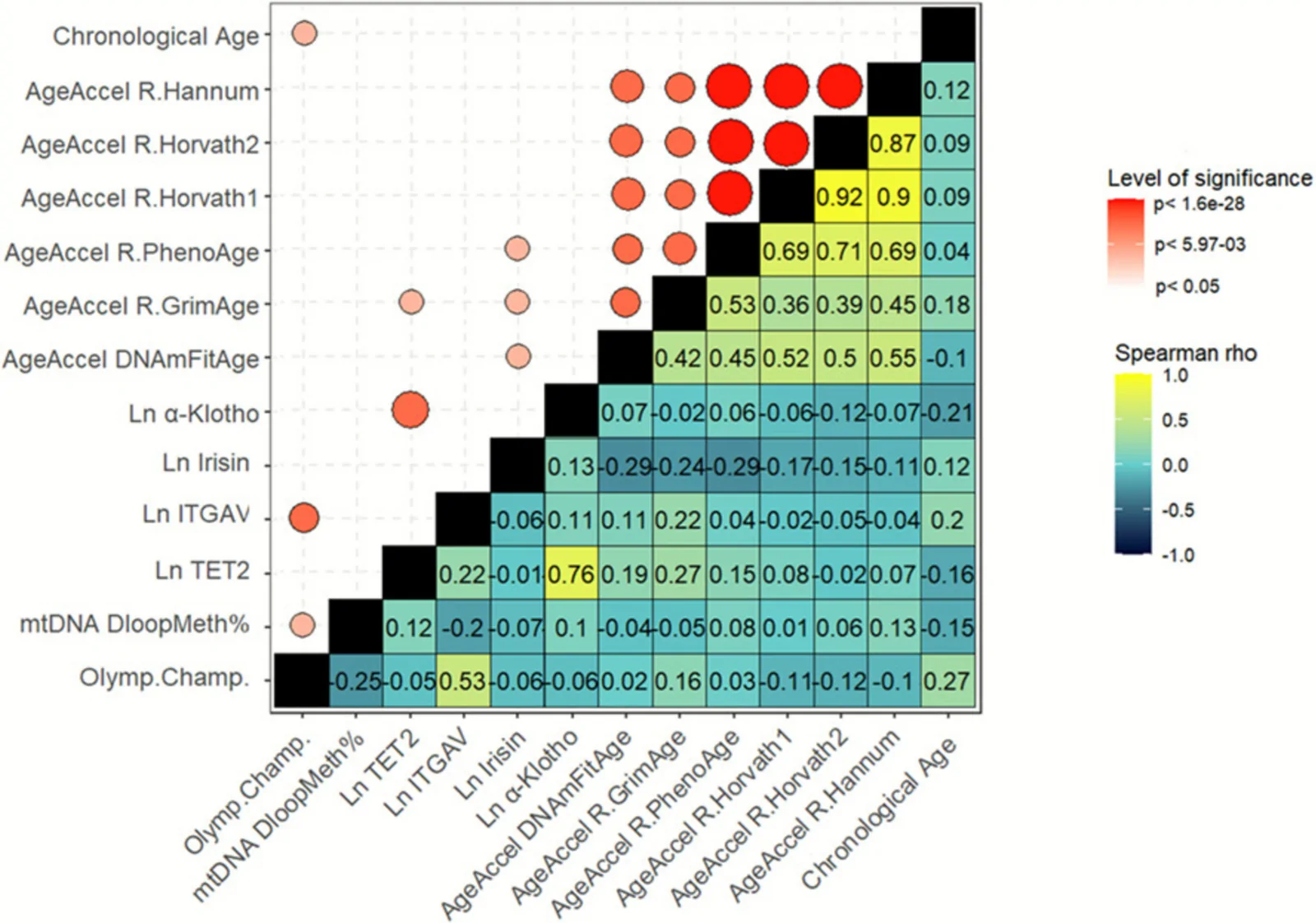
Non-parametric associations between molecular markers and biological aging estimators. The heatmap displays the relationships among the measured variables, including molecular markers, biological aging estimators, chronological age, and Olympic champion status. The lower triangle represents Spearman’s correlation coefficients (ρ), while the upper triangle shows the corresponding *p*-values, with color and size indicating the − log_10_(*p*-value)

**Fig. 3:**
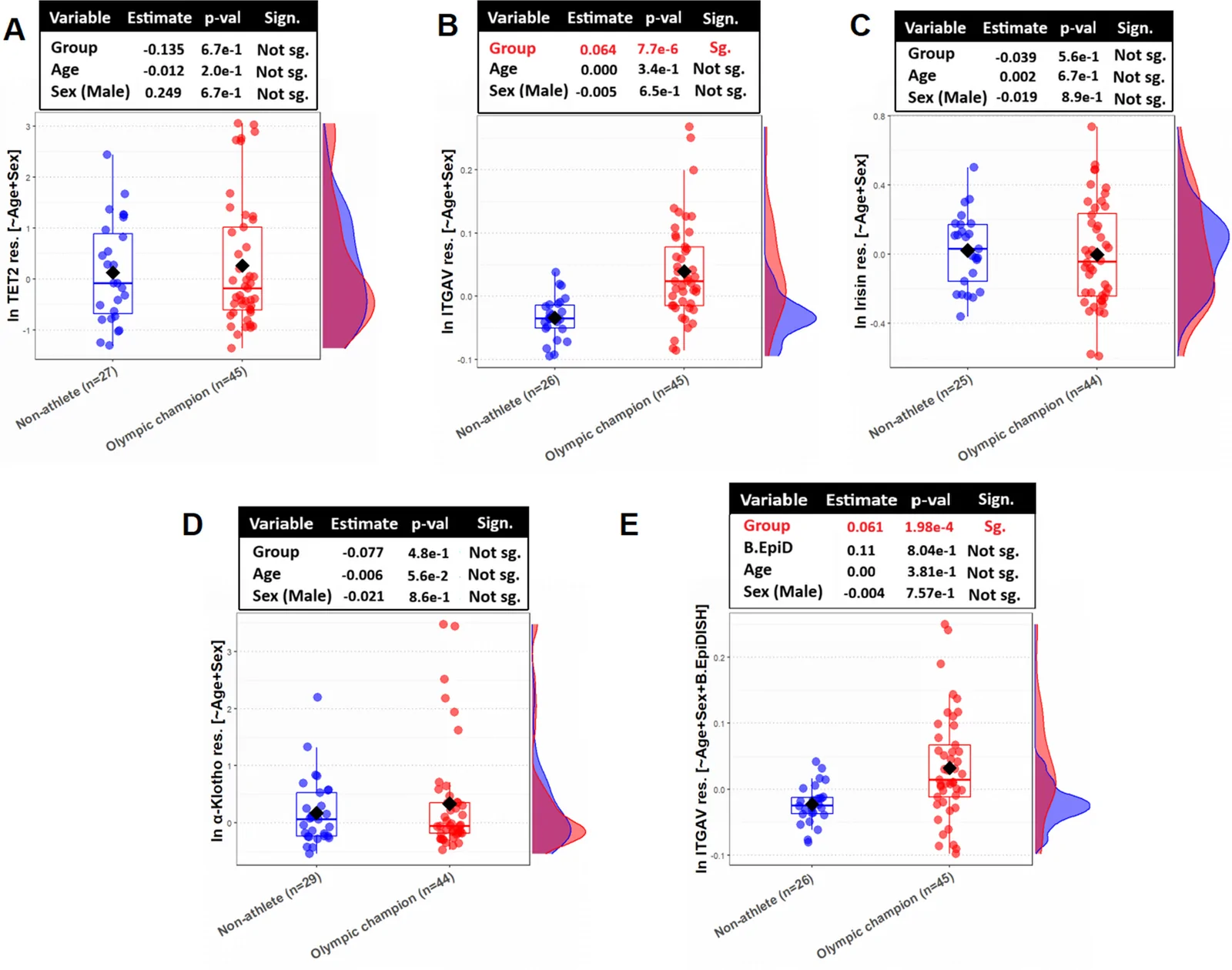
Chronological age- and sex-adjusted differences between non-athletes and Olympic champions. **A** lnTET2, **B** lnITGAV, **C** lnIrisin, **D** lnα-Klotho protein results after age and biological sex adjustments using robust linear models. **E** ITGAV results after including cell component data in the robust linear model. Blue and red dots represent covariate-adjusted residuals of the variables; black squares indicate group means. Boxes represent the interquartile range (IQR), and horizontal lines within boxes indicate medians. The distribution of each group is depicted in the side panels. Robust model estimates and corresponding *p*-values are shown above each graph, Sg.=significant effect

ITGAV was significantly higher (rho = 0.53, *p* = 1.614 × 10^–6^) in Olympic champions. In contrast, mtDNA D-loop methylation (rho = −0.25, *p* = 0.01913) was significantly lower in the Olympic champions (Fig. 2). Since age and biological sex could be potential confounders in our measurements, both variables were included in a robust linear model to elucidate the independent effect of the groups. After adjusting for age and biological sex confounders, the significant effects persisted for ln ITGAV protein content (estimate = 0.064, *p* = 7.66 × 10^–6^, Fig. 3B) and mtDNA D-loop methylation (estimate = −1.631, *p* = 0.017, Fig. 1A) variables, indicating that group differences in molecular markers are independent of chronological age and biological sex.

### The effects of blood cell composition on molecular markers

After adjusting for chronological age and biological sex, we investigated whether blood cell composition influenced the measured variables and whether it significantly contributed to the detected associations. Given that regular exercise and aging affect immune function, we examined immune cell proportions using EpiDISH software and the Houseman algorithm. The estimated B cell proportion showed a significant positive correlation with ln ITGAV and Olympic champion status (Fig. 1B). Despite this potential confounder, adjustment for sex, age, and B cell composition in group comparisons revealed that Olympic champions still exhibited significantly higher ITGAV levels (Fig. 3E).

### Associations between molecular markers and biological age estimators

**Fig. 4:**
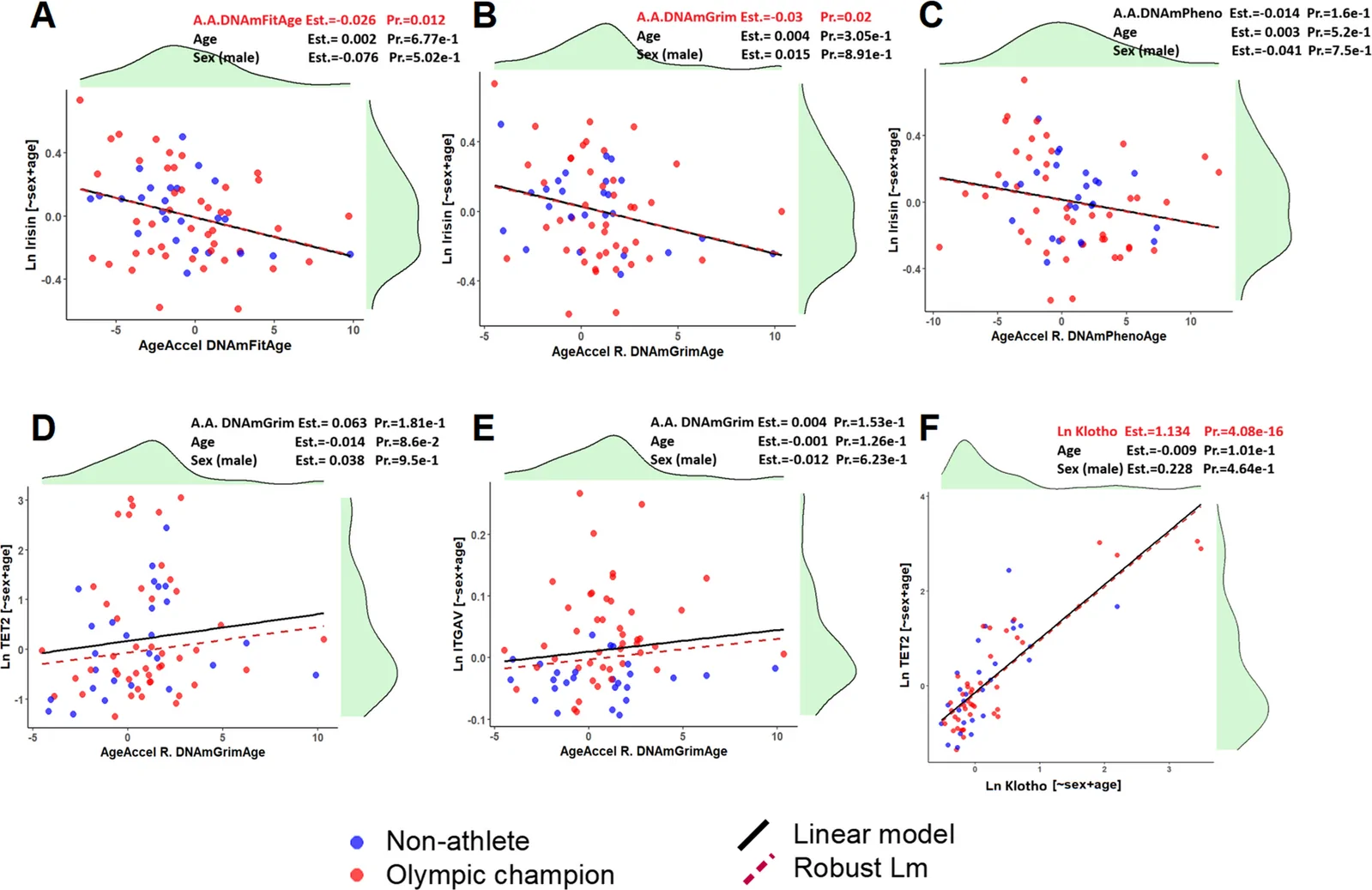
Chronological age- and sex-adjusted associations with epigenetic biomarkers of aging. Scatter plots depict associations adjusted for chronological age and biological sex using robust linear models. Adjusted associations between ln-transformed Irisin levels and A.A. DNAmFitAge (**A**), A.A.R. DNAmGrimAge (**B**), and A.A.R. DNAmPhenoAge (**C**). The adjusted associations between ln TET2 and A.A.R DNAmGrimAge is shown in panel **D**. The adjusted robust corrletion between ln-transformed ITGAV and A.A.R. DNAmGrimAge is shown in panel **E**. Panel **F** depicts the realtionship between ln-transformed TET2 and Klotho. Model stimates (Est.) and *p*-values (Pr.) are shown in the upper right corner of each scatter plot for the respective variables included in the model. The notation [∼sex + age] indicates that age- and sex-adjusted residuals are plotted to visualize the unexplained variance. Blue dots represent non-athlete individuals, while red dots depict Olympic champions. Black solid lines indicate standard linear fits, whereas dark red dashed lines represent robust linear associations

To elucidate the potential connection between selected molecular markers and biological age estimators, the Spearman correlation analysis was employed. We chose Spearman’s rho as a first-line analysis since many variables showed significant deviation from the normal distribution. Notably, we found that mtDNA D-loop methylation did not show any association with measures of epigenetic age acceleration (Fig. 2). Robust DNAm GrimAgeAcceleration showed a weak but significant negative association with Ln Serum irisin levels (rho = −0.24, *p* = 0.044) and a positive relation to Ln TET2 (rho = 0.27, *p* = 0.0202) (Fig. 2). Additionally, Ln irisin levels show a negative association with both DNAm AgeAccel Robust PhenoAge (rho = −0.29, *p* = 0.015) and AgeAccel DNAm FitAge (rho = −0.29, *p* = 0.0157) (Fig. 2). However, ln α-Klotho and Ln TET2 had a relatively strong positive correlation (rho = 0.76, *p* = 1.388 × 10^–14^). Since chronological age and biological sex may affect the circulatory level of the measured protein, and indeed, Klotho and irisin are connected to chronological age, robust linear models were applied to account for these factors, including chronological age and sex as covariates. After the adjustments, only irisin showed a significant robust association with A.A. DNAmFitAge (*Est.* = −*0.0259, p* = *0.012, *Fig. 4A*) and A.A. R. DNAmGrimAge (Est.* = −*0.0298, p* = *0.02, *Fig. 4B), indicating that irisin may contribute to slowing the ageing process independent of sex and chronological age. On the other hand, irisin association with PhenoAge was not significant (*Est.* = −*0.0137, p* = *0.16, *Fig. 4C*) after adjustments, similarly to Ln TET2 (Est.* = *0.063, p* = *0.181, *Fig. 4D*) and Ln ITGAV (Est.* = *0.004, p* = *0.153, *Fig. 4E) to A.A. R. DNAmGrimAge (Fig. 2).

One of our intriguing findings is the robust association between ln Klotho and ln TET2 levels (Fig. 4F). Although this association was attenuated after adjusting for sex and age, it remained statistically significant. Collectively, these findings suggest that while chronological age, biological sex, and blood cell composition may influence underlying biological processes, they do not entirely account for the differences observed between Olympic champions and non-athletes.

## Discussion

One of the notable findings of our investigation was that high-resolution melting analysis revealed lower overall methylation of the mtDNA D-loop region in Olympic champions compared with controls. So far, higher methylated mtDNA has been linked to diseases, such as amyotrophic lateral sclerosis [43] and cerebral autosomal dominant arteriopathy with subcortical infarcts and leukoencephalopathy [44]. On the other hand, patients with severe obesity have higher methylation levels of the mtDNA [45]. Moreover, patients with Aicardi-Goutières Syndrome (AGS) also exhibit higher methylation levels of the D-loop of mtDNA [46]. Blood-derived D-loop methylation of mtDNA and the related copy number of mtDNA, which is used to estimate the health of mitochondria [46], is questionable due to the heterogeneous cell type composition. After correction for cell type composition, only two CpG sites, located in myelin transcription factor 1-like (MYT1L) and APCDD1L-AS12 genes, retained significant associations with ITGAV levels. MYT1L and APCDD1L-AS1 play a role in transcriptional regulation, neurodevelopment, and molecular signaling pathways [47, 48]. Although these genes may reflect residual epigenetic patterns, they likely continue to contribute to the maintenance of adaptive physiological systems in Olympic champions based on the epigenetic cell memory [49, 50]. Furthermore, they can regulate ITGAV expression through epigenetic or IncRNA mechanisms [51]. Whether the hypomethylation of the D-loop is associated with exercise performance or the level of physical fitness needs to be clarified. Interestingly enough, the level of mtDNA methylation is not correlated with the measured aging markers. The lack of correlation between mtDNA and nuclear DNA methylation in Olympic champions and non-athletes suggests that they differentially influence the methylation and demethylation processes of mtDNA and nuclear DNA in these two genomic compartments. This divergence could reflect the unique structural and functional context of mtDNA, which lacks canonical chromatin organization and is subject to different enzymatic and environmental influences. This is supported by several in vitro studies, which demonstrated that mtDNA and nDNA methylation/demethylation are not associated, and they do not show correlation [52]. In addition, technical or structural factors influence the extent of mtDNA methylation; Shao et al. [53] recently demonstrated that the amount of mtDNA-5mC is much lower (< ~ 0.2 + 0.75%) than previously reported.

In addition to differences in mtDNA D-loop methylation, DNA methylation-based aging clocks showed a significant relationship with Klotho, irisin, its receptor ITGAV, as well as with epigenetic regulators such as TET (DNA demethylation). Specifically, ITGAV methylation or expression was significantly higher, and the methylation in the mtDNA D-loop region was lower in Olympic champions. The increased ITGAV expression or methylation in Olympic champions may reflect enhanced integrin-mediated signaling and cellular adhesion pathways that support skeletal muscle remodeling and facilitate the involvement of αV (encoded by ITGAV) in exercise-mediated signaling cascades [54, 55]. In addition, higher ITGAV expression may indicate mechanotransduction [56] and angiogenesis [57] in response to long-term, high-intensity training. Integrin αV is a component of several heterodimeric receptors (e.g., αVβ3, αVβ5) that mediate communication between the extracellular matrix (ECM) and cytoskeleton. Activation of these receptors during repeated muscle loading enhances downstream signaling through focal adhesion kinase (FAK), phosphoinositide 3-kinase/protein kinase B (PI3K–Akt), and (mitogen-activated protein kinase) MAPK pathways, contributing to improved muscle adaptation and recovery capacity [58, 59]. Thus, higher ITGAV expression may represent an adaptive molecular signature of superior mechanosensitivity and structural integrity in elite athletes.

Conversely, the lower mtDNA D-loop methylation observed in elite athletes could indicate enhanced mitochondrial biogenesis and transcriptional activity. The D-loop region controls mtDNA replication and transcription; its higher methylation is associated with reduced mitochondrial gene expression and impaired oxidative metabolism [4, 60]. Lower methylation levels in the mitochondrial D-loop region are inversely correlated with mitochondrial DNA copy number, suggesting that reduced methylation may enhance mitochondrial replication and gene expression [44]. Our results are similar to those reported by Ruple et al., who found the first evidence that six weeks of resistance training dynamically reduced methylation (63% of the analyzed mtDNA CpG sites) in older participants [4]. Therefore, lower D-loop methylation may facilitate greater mitochondrial gene transcription and oxidative phosphorylation, which are essential for sustained aerobic performance [61]. In this context, lower methylation of the mitochondrial control region might serve as an epigenetic marker of mitochondrial efficiency and endurance adaptation in elite athletes. Tet enzymes, including TET2, play a crucial role in DNA methylation processes [62]. Our finding that TET2 levels positively correlate with DNAm GrimAgeAcceleration aligns with studies indicating that TET2-mediated epigenetic modifications can influence aging processes, including DNA methylation changes associated with age acceleration [63]. The lack of significant association between TET2 and epigenetic age estimators after adjusting for chronological age and sex is consistent with research suggesting that these factors may modulate the relationship between TET2 and aging biomarkers [63, 64]. Although these findings highlight that TET2 involvement in nuclear DNA methylation and aging, there is no reliable evidence that TET enzymes, which convert 5mC to 5hmC in nuclear DNA, also localize or function in mitochondria [65].

We already know some potential mediators linking lower D-loop methylation with enhanced mitochondrial capacity and fitness. One of them is the exercise-induced reactive oxygen species (ROS), which can modulate epigenetic patterns, including mtDNA methylation, suggesting a link between oxidative stress and mitochondrial adaptation [66]. Intermittent hypoxia can also influence mtDNA methylation, particularly in the D-loop region, potentially altering mtDNA replication and transcription, and thus mitochondrial capacity [5, 61]. Furthermore, DNA demethylases (TET enzymes) and methyltransferases (DNMTs) may mediate mtDNA methylation changes in response to physiological stressors, providing a mechanistic pathway linking epigenetic remodeling to improved mitochondrial function. Previously described that a mitochondrial isoform of DNMT1 (mtDNMT1) translocates into mitochondria, binds mtDNA, and may methylate mitochondrial DNA [67]. In a genome-wide mtDNA methylation study in human cells, knockdown of DNMT3B (and DNMT1/DNMT3A) resulted in significantly reduced mtDNA methylation, supporting a role of DNMT isoforms in establishing or maintaining mtDNA methylation [65]. In addition, another potential mediators are the enzymes of the TET dioxygenase family (TET1, TET2, TET3), which can mediate active DNA demethylation in the nucleus, and have also been detected in mitochondria. These data suggest the possibility that demethylation machinery operates on mtDNA, not just on nuclear DNA [68]. Indeed, there is some evidence that mtDNA methylation may be dynamically regulated, similarly to nuclear DNA through of both methylation (via DNMTs) and hydroxymethylation (5‑hmC)/demethylation (via TETs) marks in mtDNA [69].

The positive correlation between α-Klotho and TET2 expression has been observed in various studies, suggesting a potential interplay between these two proteins in aging and cellular processes. TET2 activity can influence gene expression patterns, affecting the regulation of the α-Klotho gene [62]. Toan et al. (2022) [70] demonstrated in SAMP1/Klotho^−/−^ mice, TET2-mediated DNA demethylation upregulated acetylcholine receptor expression, indicating a functional link between TET2 and Klotho in aging-related processes. In human peripheral blood cells, higher Klotho expression is accompanied by increased activity of DNA methylation-related enzymes, suggesting that Klotho levels may be associated with epigenetic regulatory mechanisms, including TET2 [71]. The observed negative correlation between serum irisin levels and epigenetic age estimators (DNAm GrimAgeAcceleration, DNAm FitAge, and DNAm AgeAccel Robust PhenoAge) suggests that higher irisin concentrations are linked to a slower ageing process. These findings indicate that elevated irisin levels may be associated with a younger biological age. This relationship remained significant even after adjusting for chronological age and sex, indicating that irisin could play a role in modulating aging processes independently of these factors. Previously described that plasma irisin levels were positively correlated with telomere length in healthy adults [72]. Since telomere length is considered a marker of biological aging, this association supports our results that irisin may influence aging processes, and the physical exercise-related adaptation is related to methylation differences beneficial to aging, which may involve the role of irisin in these mechanisms [73]. By enhancing mitochondrial function, irisin may help mitigate oxidative stress and inflammation, both of which are implicated in the aging process [74–76].

An additional layer of interpretation emerges when considering the role of oxygen availability and hypoxia-related signaling in shaping the observed epigenetic patterns. Intermittent hypoxic training can significantly improve the aerobic capacity of athletes and can trigger metabolic adaptation and epigenetic processes [77, 78]. In this regard, our results might depict an adaptation away from chronic hypoxia toward enhanced oxygen-dependent metabolic efficiency, consistent with elite athletic conditioning. Previously described that intermittent hypoxia and training combination triggers physiological responses (oxidative, angiogenic) which could underlie improved oxygen transport and utilization in elite athletes, supporting the enhanced oxygen-dependent metabolic efficiency [79]. Early-life exercise has been shown to provide long-term benefits through exercise-induced epigenetic changes, and in Olympians, intensive training typically starts at an early age, reinforcing such adaptations [80]. Moreover, the training, including high-intensity exercise, unloading, and retraining, affects DNA methylation in muscle, suggesting transcriptional memory may be mediated via epigenetic modifications [81]. Transient, exercise-induced hypoxia is a well-recognized driver of mitochondrial remodeling and vascular adaptation. In this context, the markedly lower mtDNA D-loop methylation detected in Olympic champions may reflect long-term adaptation to repeated short-term hypoxic stress during high-intensity training, which promotes mitochondrial biogenesis and enhanced oxidative phosphorylation. Because hypoxia has been shown to increase D-loop methylation and suppress mitochondrial transcription, the reduced methylation seen here likely indicates superior oxygen utilization rather than chronic hypoxic exposure. This interpretation is further supported by elevated ITGAV levels, which suggest augmented angiogenic and mechanotransductive signaling that improves tissue oxygen delivery, and by increased TET2 expression, whose enzymatic activity is oxygen-dependent. The positive relationship between TET2 and Klotho also aligns with an anti-oxidative, anti-hypoxic systemic phenotype. Together, these findings suggest that the molecular signatures distinguishing Olympic champions (lower mtDNA D-loop methylation, higher ITGAV and TET2 expression, and greater Klotho levels) represent a coordinated epigenetic and metabolic adaptation that enhances mitochondrial efficiency and protects against the detrimental effects of chronic hypoxia and oxidative stress.

As a limitation, the present study cannot directly address the relationship between D-loop methylation and mtDNA copy number in this cohort. D-loop methylation was assessed using MS–HRM, and no orthogonal validation (e.g., targeted bisulfite sequencing or LC–MS–based 5-mC quantification) was performed. Therefore, confirming these findings and fully elucidating this phenomenon remain important objectives for future research. Future investigations may consider assessing these parameters in parallel to provide additional insight into their potential interplay in the context of elite athletic performance.

## Conclusion

Here, we show that methylation levels of nuclear DNA and mtDNA are likely governed by independent processes, and that lower D-loop methylation could exert yet-to-be characterized effects on health. Epigenetic aging is linked to circulating factors such as Klotho and irisin, indicating a complex regulation behind DNA methylation. Future studies incorporating functional mitochondrial assays, such as high-resolution respirometry, reactive oxygen species measurements, and mitochondrial membrane potential analyses, will be valuable to directly link D-loop methylation with mitochondrial performance. Integrating muscle biopsy-based proteomics and metabolic profiling could further elucidate the mechanistic impact of these epigenetic modifications. Longitudinal exercise interventions may provide additional insight into the translational relevance of D-loop methylation for muscle function and metabolic health.

## Supplementary Information

Below is the link to the electronic supplementary material.

Supplementary file1 (DOCX 421 kb)

## Data Availability

Data is available upon reasonable request to the corresponding author.

## Abbreviations

AGS: Aicardi-Goutières Syndrome
BDNF: Brain-derived neurotrophic factor
ECM: Extracellular matrix
EWAS: Epigenome-wide association study
FAK: Focal adhesion kinase
FDR: False discovery rate
ITGAV: Irisin receptor integrin alpha-V
MAPK: Mitogen-activated protein kinase
mtDNA: Mitochondrial DNA
MYT1L: Myelin transcription factor 1 like
nDNA: Nuclear DNA
PI3K–Akt: Phosphoinositide 3-kinase/protein kinase B
SAMP1: Spindle-associated membrane protein 1
TET2: Ten-eleven translocation enzyme 2
