# Supplementary material for: Epigenetic insights of Olympic champions: nuclear and mitochondrial DNA methylation and regulators of aging
Source: GeroScience. 2026 Jan 17;48(6):7923–38. doi: 10.1007/s11357-025-02092-9 (PMC13638895; doi:10.1007/s11357-025-02092-9)
Supplement: Supplementary file 1 [file 11357_2025_2092_MOESM1_ESM.docx]

**Supplementary Figure 1.** Age distribution of the subjects by biological sex and group.

**Supplementary Figure 2**. Answer distribution of the subjects depicting their chronic disease status, medicine use, and smoking habits.

**Supplementary Figure 3.** Distribution of sports and years since career termination among Olympic champions. (A) The number of Olympic champions in each sport discipline. (B) Jitter plot showing the number of years since athletes ended their professional sports career. Blue points represent retired athletes, while red points represent currently active athletes. A black horizontal bar denotes the mean number of years since retirement.

**Supplementary Figure 4.** Gene set enrichment results adjusted for probe numbers. Blue ribbons represent processes from the Gene Ontology category Biological Process (BP), green ribbons indicate Molecular Function (MF) terms, and orange ribbons correspond to Cellular Component (CC) terms. The length of each ribbon represents the –log₁₀(p-value).
